# 7-Dehydrocholesterol protects against circadian disruption and experimental colitis: potential role of RORα/γ

**DOI:** 10.1093/lifemeta/load034

**Published:** 2023-09-19

**Authors:** Feng Li, Shubin Lin, Zhiyi Tan, Yanqing Pang, Shuai Wang

**Affiliations:** Guangzhou Eighth People’s Hospital, Guangzhou Medical University, Guangzhou, Guangdong 510440, China; Institute of Molecular Rhythm and Metabolism, Guangzhou University of Chinese Medicine, Guangzhou, Guangdong 510006, China; Guangzhou Customs Technology Center, Guangzhou, Guangdong 510623, China; Department of Phase I Clinical Research Center, The Second Affiliated Hospital of Guangzhou University of Chinese Medicine (Guangdong Provincial Hospital of Chinese Medicine), Guangzhou, Guangdong 510006, China; Institute of Molecular Rhythm and Metabolism, Guangzhou University of Chinese Medicine, Guangzhou, Guangdong 510006, China

## Dear Editor,

Circadian rhythms are physical, behavioral, and mental changes that follow a roughly 24-h cycle. Our prior researches have established a significant correlation between the disruption of the circadian rhythm and the severity of experimental colitis, an animal model replicating inflammatory bowel disease (IBD) in humans [1, 2]. IBD is a chronic inflammatory disease of the gastrointestinal tract and is divided into ulcerative colitis and Crohn’s disease. Approximately 0.5% of the population in the Western world suffers from IBD [3]. Currently, commonly used drugs for IBD include aminosalicylates, glucocorticoids, immunomodulating drugs, and biological agents. Nonetheless, these therapeutics exhibit restricted efficacy and are frequently accompanied by a pletho­ra of adverse effects [4]. Consequently, it is imperative to explore novel compounds targeting IBD as potential alternatives. In this context, gut microbiota-derived metabolites, which possess the ability to modulate circadian rhythms, emerge as highly promising lead compounds for the prevention and management of IBD.

**Figure 1:**
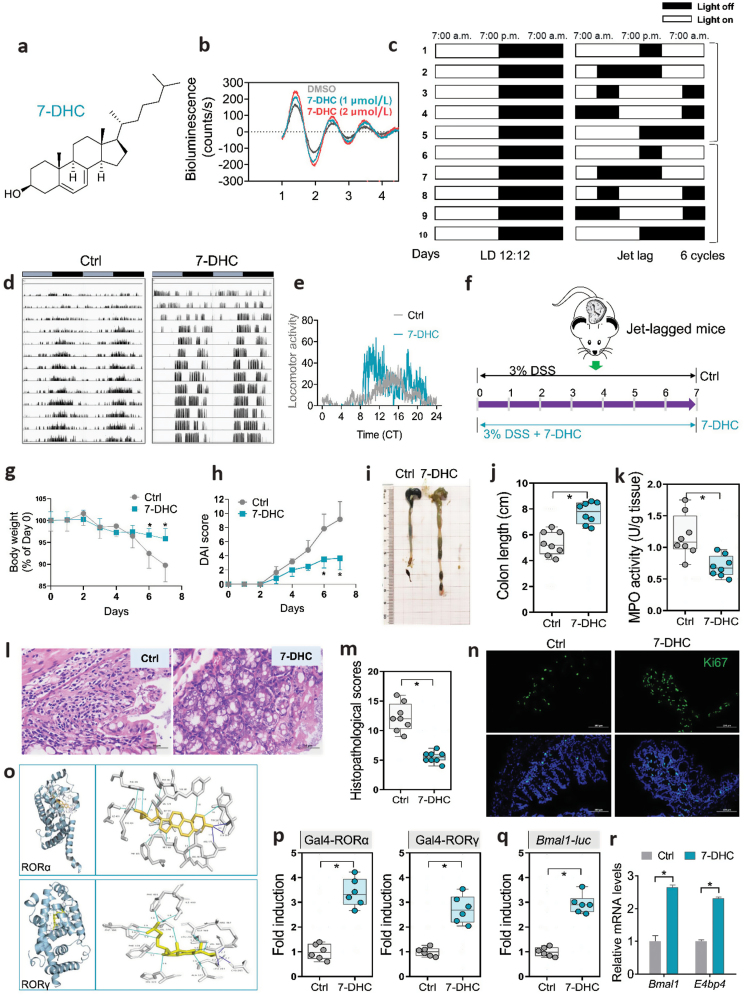
7-DHC ameliorates experimental colitis through RORα/γ activation and circadian restoration. (a) Chemical structure of 7-DHC. (b) Bioluminescent recordings of BMAL1::Luc U2OS cells treated with 7-DHC or vehicle. (c) Experimental scheme for the establishment of the jet-lagged mouse model. (d) Representative double-plotted actograms for daily locomotor activities of control (Ctrl, left panel) and 7-DHC (right panel) mice in constant dark. Horizontal gray and black bars at the top of each actogram represent subjective light and dark phases. *n* = 6 for each group. (e) Locomotor activity was monitored for 7-DHC and Ctrl mice starting from day 5 to day 13. CT, circadian time. (f) Experimental design of jet-lagged colitis mice with 7-DHC treatment. The mice were fed with 3% DSS and treated with 7-DHC or vehicle for 7 days. (g) Weight loss, (h) DAI, (i) Colon image, (j) Colon length of 7-DHC-treated and Ctrl mice with circadian disruption. Data are mean ± SD (*n* = 8). ^*^P < 0.05 (Student’s *t* test). (k) Myeloperoxidase (MPO) activities of 7-DHC-treated and Ctrl mice. Data are mean ± SD (*n* = 8). ^*^P < 0.05 (Student’s *t* test). (l) Representative images of hematoxylin and eosin staining in the colon. Scale bars, 50 μm. (m) Histopathological scores of 7-DHC-treated and Ctrl mice. Data are mean ± SD (*n* = 8). ^*^P < 0.05 (Student’s *t* test). (n) Immunofluorescence for Ki67 in colons of 7-DHC-treated and Ctrl mice. Scale bars, 100 μm. (o) Molecular docking shows the binding interaction of 7-DHC with RORα (up panel) and RORγ (down panel) based on the binding energy. (p) Luciferase reporter assays show that 7-DHC increases Gal4-RORα (left panel) and Gal4-RORγ (right panel) activity in HEK293T cells. Data are mean ± SD (*n* = 6). ^*^P < 0.05 (Student’s *t* test). (q) Luciferase reporter assays show that 7-DHC increases *Bmal1-luc* transcription in HEK293T cells. Data are mean ± SD (*n* = 6). ^*^P < 0.05 (Student’s *t* test). (r) qPCR analyses of *Bmal1* and *E4bp4* expressions in colons of 7-DHC-treated and Ctrl mice with colitis. Data are mean ± SD (*n* = 6). ^*^P < 0.05 (Student’s *t* test).

We postulated that the utilization of molecules that enhance circadian rhythm may hold potential benefits for the management of IBD. We carried out a screening of circadian rhythm-enhancing molecules from a library of gut microbiota-derived metabolites using the U2OS cells stably expressing the BMAL1::Luc luciferase reporter, which exhibit sustained circadian rhythms *in vitro*. In order to assess the clock amplitude-enhancing properties, an initial investigation was conducted with 20 compounds in the library (Supplementary Fig. S1a). Notably, 7-dehydrocholesterol (7-DHC, depicted in Fig. 1a), which is a cholesterol derivative produced by the gut microbiota, exhibited a dose-dependent effect in augmenting the amplitude of the BMAL1::Luc reporter rhythm (Fig. 1b and Supplementary Fig. S1b). To further investigate whether 7-DHC could restore circadian dysfunction in autonomous activities, we established a circadian rhythm-disrupted mouse model using a jet lag protocol [1] (Fig. 1c). Jet lag induced circadian dysfunction in the wheel-running activity of mice (Fig. 1d). The jet-lagged mice were then treated with 7-DHC or vehicle under constant darkness. Though the disrupted circadian rhythm in the wheel-running activity of 7-DHC treated mice was not improved in the first 4 days, this compound showed a significant increase in wheel-running activity in the resting phase and a decrease in the active phase starting from day 5 to day 13 (Fig. 1d and e, Supplementary Fig. S2). These findings indicate that 7-DHC is a circadian rhythm-enhancing molecule, and could reverse circadian disruption in locomotor activity.

We previously found that jet lag-induced circadian disruption exacerbated experimental colitis in mice [2, 3]. Considering the role of 7-DHC as a circadian rhythm modulator, we subsequently evaluated its potential to mitigate the severity of colitis. First, a dextran sulfate sodium (DSS)-induced colitis mouse model with circadian dysfunction was established with jet lag (Fig. 1f). The colitis mice with circadian disruption were simultaneously treated by gavage with 50 mg/kg 7-DHC or vehicle at circadian time (CT) 0. Compared with the controls, 7-DHC-treated colitis mice showed less body weight loss, lower disease activity index (DAI) scores, longer colons, and lower myeloperoxidase activity, indicating that 7-DHC alleviated experimental colitis with circadian disruption (Fig. 1g–k). Less severe colitis in 7-DHC-treated mice was confirmed by histological examinations as evidenced by less severe neutrophil infiltration, less extensive mucosal sloughing, and fewer ulcers (Fig. 1l and m). Consistently, the fluorescence intensity of Ki-67 (a proliferation marker) in epithelial cells of the colonic mucosa was significantly increased in 7-DHC-treated mice (Fig. 1n). Furthermore, an investigation was carried out to examine the potential anti-colitis properties of 7-DHC in mice unaffected by circadian disruption. Notably, 7-DHC exhibited a moderate anti-colitis effect in mice with an intact circadian rhythm, as evidenced by the results depicted in Supplementary Fig. S3. Overall, circadian disruption sensitizes mice to DSS-induced colitis, while 7-DHC potentially ameliorates experimental colitis in mice by affecting the circadian rhythm.

Circadian rhythm is tightly controlled by a circadian clock system consisting of several clock proteins including retinoic acid receptor-related orphan receptors (RORs), brain and muscle ARNT-like 1 (BMAL1), and E4 promoter-binding protein 4 (E4BP4, also known as Nfil3) [5]. We speculated that 7-DHC could target clock proteins to modulate circadian rhythms. To further clarify the mechanism underlying the circadian rhythm-enhancing effects of 7-DHC, we performed molecular docking of 7-DHC to clock proteins. The docking result indicated that 7-DHC bound to RORα and RORγ with low free energy of < −10 kcal/mol (Fig. 1o and Supplementary Table S1). There were 3 residues involved in hydrogen bonds and 12 in hydrophobic interactions for 7-DHC with RORα or RORγ (Supplementary Tables S2–5). In the Gal4 cotransfection assay, the DNA-binding domain (DBD) of Gal4 was fused to the ligand-binding domain of RORα or RORγ. 7-DHC enhanced the transcriptional activation of RORα/γ in HEK293 cells (Fig. 1p). Supporting this finding, 7-DHC also enhanced the transcriptional activity in the *Bmal1* (a direct target gene of RORs) reporter gene (*Bmal1-luc*) in the luciferase reporter assay (Fig. 1q). Furthermore, 7-DHC significantly increased the expression of RORα/γ target genes in the colon of jet-lagged mice with colitis, suggesting that targeting RORα/γ by 7-DHC reversed circadian dysregulation in colitis (Fig. 1r). Overall, 7-DHC ameliorates experimental colitis through RORα/γ activation and circadian restoration.

Recent studies have aimed to develop candidate drug molecules targeting core components or molecular clocks such as RORs [6]. RORα/γ is widely expressed in various tissues, while RORβ is mainly expressed in the central nervous system [7]. A previous study identified hydroxycholesterol acting as ligands of RORγ [7]. Although no significant differences in affinity with RORγ were observed between 7-DHC and 25-hydroxycholesterol (Supplementary Fig. S4), it is noteworthy that 7-DHC exhibits activation of both RORα and RORγ, suggesting a broader impact of 7-DHC on circadian rhythms. Circadian rhythm-modulating mole-cules show the special merit of regulating precise and reversible circadian rhythm. 7-DHC is a circadian amplitude enhancer, alleviating colitis in two ways that are intertwined: direct activation of RORα/γ and restoration of the circadian rhythm. Previous findings indicated that functions of RORα-dependent group 3 innate lymphoid cells (ILC3s) are pivotal in mediating IBD [8]. RORγ regulates the frequency of T helper 17 (Th17) cells and controls the secretion of the cytokines interleukin (IL)-17a and IL-17f [9]. Considering the therapeutic potential of 7-DHC, it is imperative to further investigate its influence on RORα/γ, possibly mediated by the intestinal microbiota and mucosal immunity.

7-DHC is a precursor of vitamin D, and vitamin D signaling is known to play a crucial role in regulating intestinal homeostasis [10]. To decipher the role of 7-DHC in regulating intestinal homeostasis is independent of vitamin D or not, we first examined the expression levels of target genes under the influence of the vitamin D receptor. No significant alterations in these genes (i.e., *CAMP* and *Cyp3a11*) in the colons of colitis mice were observed upon 7-DHC administration, indicating a vitamin D-independent mechanism for 7-DHC (Supplementary Fig. S5). We also investigated the effect of the time dependence of 7-DHC administration on colitis. Notably, we observed an attenuation of the anti-colitis effect of 7-DHC at CT8 (when protein expression of RORγ is relatively low) compared with CT0 (when RORγ expression is relatively high) (Supplementary Fig. S6). These findings provide evidence for a direct regulatory role of 7-DHC on RORα/γ.

In summary, our findings suggest that 7-DHC could hold significant promise as a potential therapeutic agent in combating circadian disruptions and IBD. This knowledge enhances our understanding of the strength of molecules with circadian rhythm-modulating function, thereby facilitating further research and development of novel IBD therapeutics.

## Supplementary Material

load034_suppl_Supplementary_Material

## Data Availability

The online version of this article contains supplementary material, which is available to authorized users.
