# Supplementary material for: 7-Dehydrocholesterol protects against circadian disruption and experimental colitis: potential role of RORα/γ
Source: Life Metab. 2023 Sep 19;2(6):load034. doi: 10.1093/lifemeta/load034 (PMC11748971; doi:10.1093/lifemeta/load034)
Supplement: load034_suppl_Supplementary_Material [file load034_suppl_Supplementary_Material.pdf]

Supplementary Materials for

7-Dehydrocholesterol protects against circadian disruption and experimental colitis: potential role of RORα/γ

Supplementary Methods

Materials

Dextran sulfate sodium (DSS) was procured from MP Biomedicals (Santa Ana, CA, USA). Fetal bovine serum and trypsin were obtained from Hyclone (Logan, UT, USA). TRIzol reagent, reverse transcriptase mix, and SYBR Green Master Mix were acquired from Vazyme (Nanjing, Jiangsu, China). The Myeloperoxidase (MPO) kit was purchased from Jiancheng Institute of Biotechnology (Nanjing, Jiangsu, China). The transfection reagent jetPRIME was sourced from Polyplus Transfection (Illkirch, France). Gal4-RORα LBD, Gal4-RORγ LBD, and *Bmal1*-luc constructs were obtained from Transheep Technologies (Shanghai, China). The Dual Luciferase Assay Kit was purchased from Promega (Beijing, China). All compounds were purchased from TargetMol (Wellesley Hills, MA, USA). The Ki67 antibody was sourced from Servicebio (Wuhan, Hubei, China).

Mice

Wild-type C57BL/6 mice were procured from HFK Biotech (Beijing, China). The mice were housed in a controlled environment with a 12-hour light and 12-hour dark cycle, and provided with *ad libitum* access to food and water. For colitis induction, male or female mice aged 8 to 12 weeks were used. All experimental procedures involving mice were conducted in compliance with the guidelines and approval of the Institutional Animal Care and Use Committee of Guangzhou University of Chinese Medicine.

Colitis induction

To induce colitis, we dissolved DSS in drinking water to create a 3% (w/v) concentration, and mice were exposed to this solution for 7 consecutive days. To assess the impact of 7-DHC on colitis, mice were administrated orally with 7-DHC at a dosage of 50 mg/kg daily at CT0 or CT8 for a week before initiating DSS treatment. The administration of 7-DHC was consistently maintained throughout the DSS treatment period. To assess the clinical progression of colitis, we evaluated the disease activity index (DAI) daily. The DAI encompasses a combination of scores for weight loss compared to the initial weight, stool consistency, and occult blood presence. Each parameter was assigned a score ranging from 1 to 4, with a maximum total score of 12. On day 6, we collected mouse colon samples for further investigations. To conduct hematoxylin & eosin (H&E) staining and scoring, we formalin-fixed and embedded the tissues in paraffin. Subsequently, 5 μm sections of paraffin-embedded tissues were subjected to H&E staining and evaluated under a light microscope. Histological damage was blindly assessed based on parameters such as mucosa thickening, ulcers, inflammatory cell infiltration, goblet cell loss, submucosa cell infiltration, and crypt abscesses. Each parameter was assigned a score ranging from 1 to 3 or 1 to 4, resulting in a maximum total score of 20.

Locomotor activity analysis

Mice were housed individually in running wheel cages (Lafayette Instrument, Lafayette, IN), which were positioned within light-tight cabinets under a 12-hour light/dark cycle. Following acclimation to the experimental setup, the mice underwent a continuous

recording period. This recording period consisted of 13 days under under constant darkness. The analysis of locomotor activity was conducted using the ClockLab software (Actimetrics, Wilmette, IL). The data obtained from the recordings were sorted, pooled, and averaged based on the corresponding light and dark cycles.

Immunofluorescence staining

Colon tissues were initially fixed in a 4% paraformaldehyde solution, followed by sequential immersion in sucrose solutions of increasing concentrations (10%, 20%, and 30%) for optimal tissue preservation. Subsequently, sections with a thickness of 6 μm were prepared from the fixed colon tissues. The sections were subjected to blocking with a solution containing 5% BSA and 0.5% Triton X-100 in phosphate-buffered saline (PBS). Following this blocking step, the sections were incubated with an anti-Ki67 antibody, a marker commonly used to identify proliferating cells. After the primary antibody incubation, the sections underwent thorough washing with PBS to remove any unbound antibodies. Subsequently, the sections were exposed to an Alexa Fluor 488-conjugated anti-rabbit secondary antibody. Finally, post-washing with PBS, the sections were mounted and subjected to imaging utilizing a laser scanning microscope (Carl Zeiss, Oberkochen, Germany).

Quantitative polymerase chain reaction (qPCR)

Total RNA was extracted from the mouse colon with TRIzol reagent following the manufacturer's instructions. cDNAs were synthesized from total RNA using a reverse transcriptase mix. The expression level of genes was determined with specific primers (provided in Supplementary Table S6) with the $2^{-\Delta\Delta Ct}$ method. *18s rRNA* was used as an internal control.

Cell treatment and luciferase reporter assay

HEK293T cells were cultured and seeded into 48-well plates, following which they were transfected with a total of 200 ng of plasmids, including Gal4-RORα/γ LBD, PGL4.35, or *Bmal1-luc*. Additionally, 10 ng of the pRL-TK vector, serving as an internal control with renilla luciferase, was co-transfected. The transfection process was conducted for 24 h to allow for optimal expression. In the subsequent day, the culture medium was replaced with phenol-free Dulbecco's Modified Eagle Medium (DMEM), either with or without the addition of 7-DHC. On the next day, the transfected cells were harvested, and their luciferase activities were quantified using the Dual-Luciferase Reporter Assay system and GloMax 20/20 Luminometer, enabling accurate measurement of luciferase levels.

Molecular docking

The 3D structure of the target protein was obtained from the Protein Data Bank (PDB) database (http://www1.rcsb.org/). Subsequently, the protein was processed using PyMOL software (www.pymol.org) to prepare it for molecular docking. This involved tasks such as dehydrating the protein and hydrogenating its atoms. For molecular docking, AutoDockTools 1.5.6 and AutoDock Vina software were employed. The binding free energy was utilized as the evaluation criterion to assess the strength of the interaction between the protein and the compound. Lower energy values indicate a more favorable binding affinity, increasing the likelihood of the occurrence of the compound-protein complex. Thus, lower energy values are considered to yield more reliable docking results. The PLIP online platform (https://plip-tool.biotec.tu-

dresden.de/) was used to analyze the interaction between molecular docking results and proteins.

Statistical analyses

Data are recorded as mean ± standard deviation (SD). The means of two groups were compared using Student's *t*-test. One-way or two-way analysis of variance (ANOVA) was performed to compare the means of more than two groups, followed by post-hoc Bonferroni test for further group comparisons. The level of significance was set at $P < 0.05$ (*).

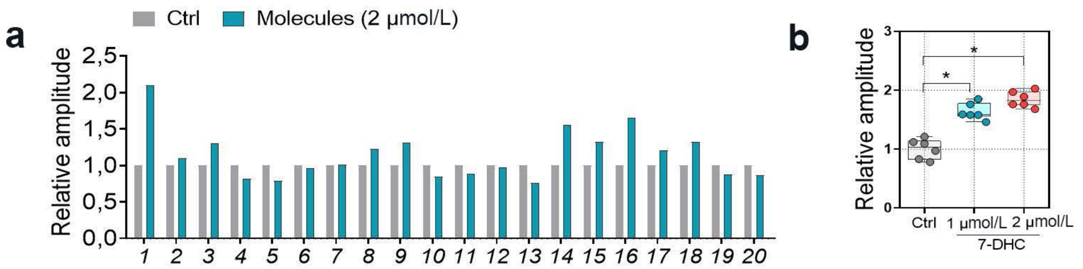

1	2	3	4	5	6	7	8	9	10	11	12	13	14	15	16	17	18	19	20
7-Dehydrocholesterol	Phosphonoacetic acid	DL-Homocysteine	3-Ureidopropionic acid	4-Guanidinobutyric acid	5'-Deoxyadenosine	4-Quinolinecarboxylic acid	Lumazine	(L)-Dehydroascorbic acid	3-(2-Hydroxyphenyl)propionic acid	6-Hydroxypyridine-3-carboxylic acid	L-Tryptophanamide hydrochloride	Dihydrouracil	12-Hydroxydodecanoic acid	Arachidic acid	Nervonic acid	Methylmalonic acid	3-(2-Hydroxyethyl)indole	2-Deoxy-D-ribose	Pipecolinic acid

Supplementary Figure S1 The effects of 20 molecules on the amplitude of the BMAL1::Luc reporter rhythm. (a) Relative amplitude of 20 molecules from the TargetMol library. (b) Relative amplitude of 7-DHC.

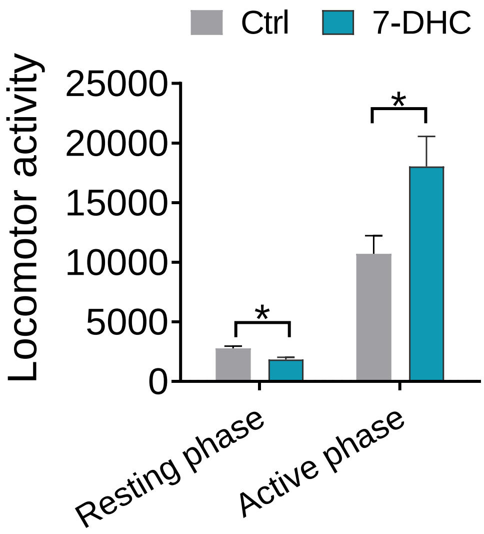

Supplementary Figure S2 Locomotor activity of the 7-DHC-treated and control (Ctrl) mice during resting phase and active phase (related to Fig. 2e).

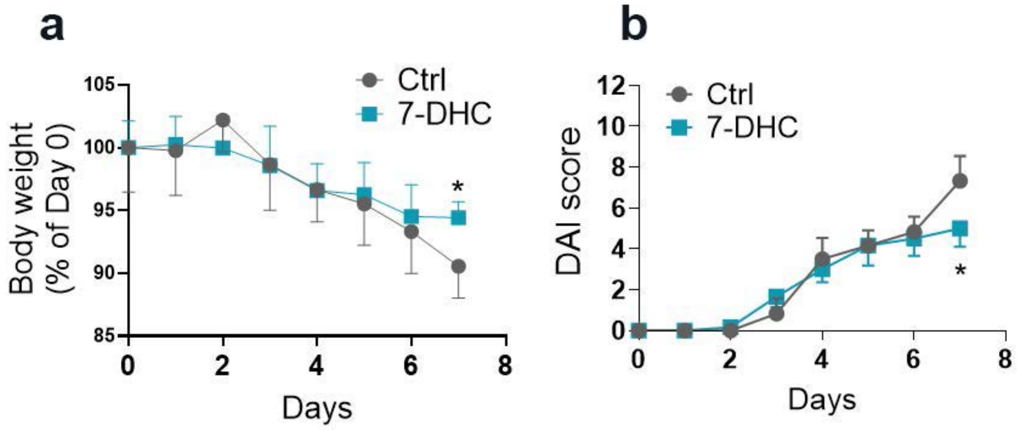

Supplementary Figure S3 The effects of 7-DHC on DSS-induced colitis mice with normal circadian rhythm. (a) Weight loss and (b) DAI of 7-DHC-treated and Ctrl mice.

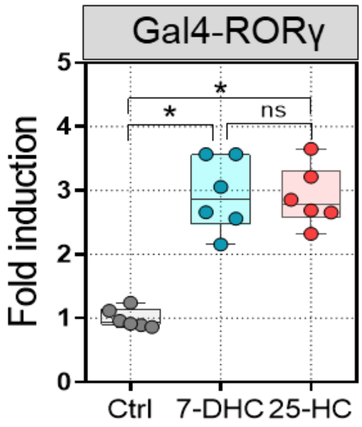

Supplementary Figure S4 The Luciferase reporter assays show that 7-DHC and 25-hydroxycholesterol (25-HC) increases Gal4-RORγ activity in HEK293T cells. Data are mean ± SD (n = 6). $^{*}P < 0.05$ (One-way ANOVA).

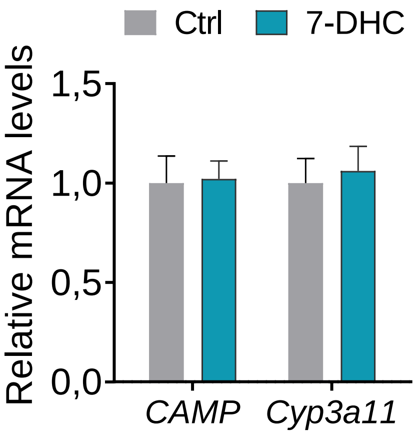

Supplementary Figure S5 The effects of 7-DHC on the expression of vitamin D receptor target genes.

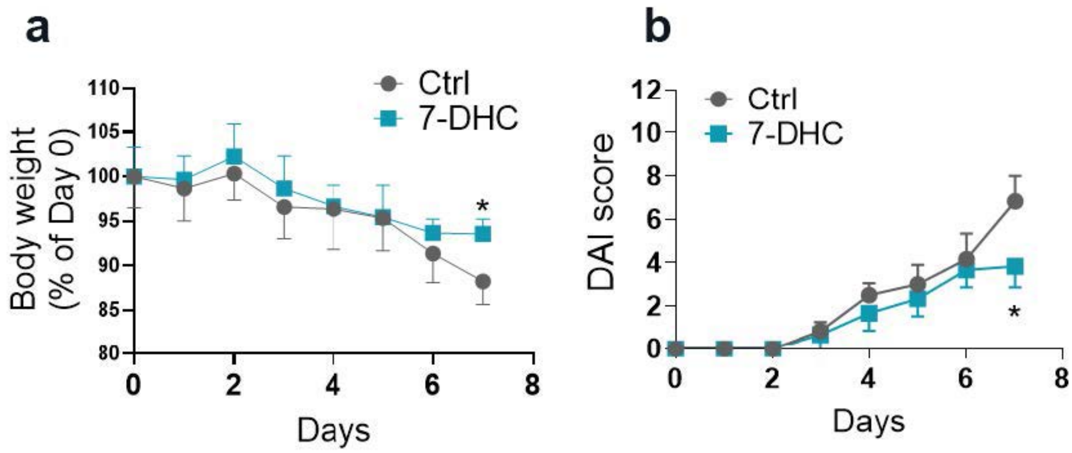

Supplementary Figure S6 The effects of 7-DHC on DSS-induced colitis mice with circadian disruption. 7-DHC was administrated at CT8. (a) Weight loss and (b) DAI of 7-DHC-treated and Ctrl mice.

Supplementary Table S1 Binding affinity of 7-DHC and RORα/γ using molecular docking.

Models	Affinity (kcal/mol)	
	RORα	RORγ
1	-11.1	-11.1
2	-11.0	-10.6
3	-10.9	-10.4
4	-9.8	-10.3
5	-9.7	-10.2
6	-9.4	-10.1
7	-8.7	-10.0
8	-8.5	-9.9
9	/	-9.6

Supplementary Table S2 Hydrophobic interactions of RORα and 7-DHC.

Index	Residue	AA	Distance	Ligand Atom	Protein Atom
1	290A	TYR	3.61	18	268
2	327A	ILE	3.57	22	599
3	365A	PHE	4.00	8	901
4	367A	ARG	3.76	16	916
5	368A	MET	3.56	6	927
6	379A	VAL	3.55	26	1015
7	380A	TYR	3.85	13	1024
8	381A	PHE	3.59	21	1035
9	391A	PHE	3.85	25	1116
10	391A	PHE	3.72	27	1114
11	400A	ILE	3.45	27	1185
12	404A	PHE	3.46	27	1218

Supplementary Table S3 Hydrogen bonds of RORα and 7-DHC.

Index	Residue	AA	Distance H-A	Distance D-A	Donor Angle	Donor Atom	Acceptor Atom
1	289A	GLN	3.65	4.05	106.71	253 [Nam]	28 [O3]
2	290A	TYR	2.45	3.28	142.00	262 [Nam]	28 [O3]
3	370A	ARG	2.54	3.28	132.12	946 [Ng+]	28 [O3]

Supplementary Table S4 Hydrophobic interactions of RORγ and 7-DHC.

Index	Residue	AA	Distance	Ligand Atom	Protein Atom
1	292A	LEU	3.65	17	254
2	323A	HIS	3.60	8	528
3	327A	ALA	3.93	4	562
4	361A	VAL	3.84	10	828
5	368A	ALA	3.38	15	885
6	376A	VAL	4.00	11	951
7	378A	PHE	3.16	8	969
8	388A	PHE	3.89	25	1049
9	388A	PHE	3.44	23	1051
10	388A	PHE	3.65	27	1046
11	400A	ILE	3.78	26	1135
12	401A	PHE	3.59	26	1145

Supplementary Table S5 Hydrogen bonds of RORγ and 7-DHC.

Index	Residue	AA	Distance H-A	Distance D-A	Donor Angle	Donor Atom	Acceptor Atom
1	285A	CYS	1.81	2.69	149.53	28 [O3]	192 [O3]
2	287A	LEU	2.38	3.18	138.52	204 [Nam]	28 [O3]
3	367A	ARG	2.19	2.69	109.98	879 [Ng+]	28 [O3]

Supplementary Table S6 Primers used for qPCR.

	Forward (5'-3' sequence)	Reverse (5'-3' sequence)
CAMP	GCTGTGGCGGTCACTATCAC	TGTCTAGGGACTGCTGGTTGA
Cyp3a11	GTGCTCCTAGCAATCAGCTT	CAGTGCCTAAAAATGGCAGAGG
Bmal1	CTCCAGGAGGCAAGAAGATTC	ATAGTCCAGTGGAAGGAATG
E4bp4	CTTTCAGGACTACCAGACATCCAA	GATGCAACTTCCGGCTACCA
18s rRNA	CGGACAGGATTGACAGATTGATAGC	TGCCAGAGTCTCGTTCGTTATCG
